# EPR Spin-Trapping for Monitoring Temporal Dynamics of Singlet Oxygen during Photoprotection in Photosynthesis

**DOI:** 10.1021/acs.biochem.4c00028

**Published:** 2024-04-29

**Authors:** Collin J. Steen, Jens Niklas, Oleg G. Poluektov, Richard D. Schaller, Graham R. Fleming, Lisa M. Utschig

**Affiliations:** † Department of Chemistry, University of California, Berkeley, California 94720, United States; ‡ Molecular Biophysics and Integrated Bioimaging Division, Lawrence Berkeley National Laboratory, Berkeley, California 94720, United States; § Chemical Sciences and Engineering Division, Argonne National Laboratory, Lemont, Illinois 60439, United States; ∥ Center for Nanoscale Materials, Argonne National Laboratory, Lemont, Illinois 60439, United States

## Abstract

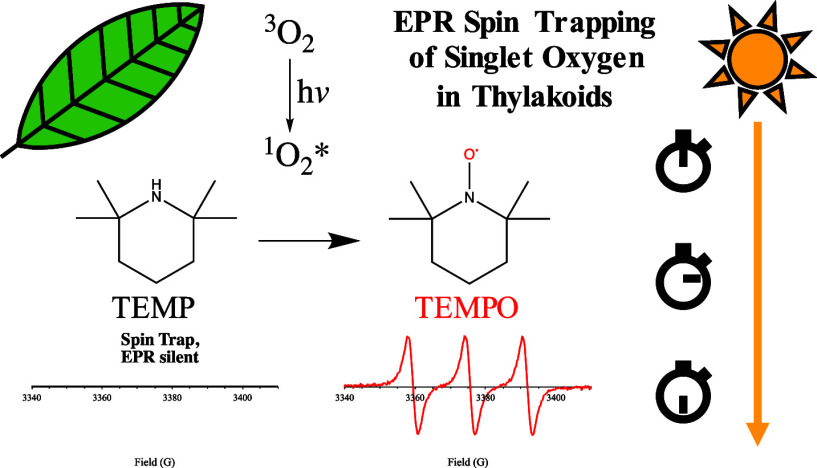

A central goal of photoprotective energy dissipation processes is the regulation of singlet oxygen (^1^O_2_*) and reactive oxygen species in the photosynthetic apparatus. Despite the involvement of ^1^O_2_* in photodamage and cell signaling, few studies directly correlate ^1^O_2_* formation to nonphotochemical quenching (NPQ) or lack thereof. Here, we combine spin-trapping electron paramagnetic resonance (EPR) and time-resolved fluorescence spectroscopies to track in real time the involvement of ^1^O_2_* during photoprotection in plant thylakoid membranes. The EPR spin-trapping method for detection of ^1^O_2_* was first optimized for photosensitization in dye-based chemical systems and then used to establish methods for monitoring the temporal dynamics of ^1^O_2_* in chlorophyll-containing photosynthetic membranes. We find that the apparent ^1^O_2_* concentration in membranes changes throughout a 1 h period of continuous illumination. During an initial response to high light intensity, the concentration of ^1^O_2_* decreased in parallel with a decrease in the chlorophyll fluorescence lifetime via NPQ. Treatment of membranes with nigericin, an uncoupler of the transmembrane proton gradient, delayed the activation of NPQ and the associated quenching of ^1^O_2_* during high light. Upon saturation of NPQ, the concentration of ^1^O_2_* increased in both untreated and nigericin-treated membranes, reflecting the utility of excess energy dissipation in mitigating photooxidative stress in the short term (i.e., the initial ∼10 min of high light).

## Introduction

As sessile organisms, plants must acclimate to a broad variety of environmental conditions, especially in response to rapid changes in incident light intensity reaching the pigment–protein complexes housed in the thylakoid membrane. In the natural environment, plants regularly encounter high light (HL) intensities, leading to the closure of reaction centers and subsequent accumulation of excited states of chlorophyll (Chl) pigments throughout the light-harvesting antenna of photosystem II (PSII). Under HL stress, singlet oxygen (^1^O_2_*), the electronically excited state of molecular oxygen, can be produced via photosensitization,^1^ a process in which a light-absorbing molecule (e.g., Chl) transfers excitation energy to ground state triplet ^3^O_2_.^2,3^ Once produced, ^1^O_2_* is an electrophilic species that is highly reactive for a wide array of biomolecules, including the D1 protein of PSII,^4−7^ photosynthetic antenna proteins,^8,9^ amino acid residues of other proteins,^10^ DNA,^11^ and lipids.^12,13^ All photosynthetic organisms thus employ a range of strategies to carefully regulate the photophysics and photochemistry of Chl excited states and the accompanying production of reactive oxygen species (ROS) such as ^1^O_2_*. For reviews on the roles of ^1^O_2_*, ROS, and oxidative stress in photosynthesis, see refs (14−20).

One important regulatory process is nonphotochemical quenching (NPQ),^21,22^ which harmlessly dissipates excess excitation energy in the light-harvesting antenna as thermal energy and therefore protects PSII against damage. Despite the known importance of NPQ-related dissipation to plant fitness and survival,^23^ many of its underlying molecular mechanisms remain controversial. Additionally, while the necessity of NPQ is frequently framed in terms of preventing the buildup of ROS, such as ^1^O_2_*, this has not been explicitly demonstrated, and more recent thinking posits that ROS play essential roles in plant signaling and stress responses.^19,24,25^ There remains much to be learned about the production, reactivity, and regulation of ROS inside intact photosynthetic systems, such as thylakoid membranes, chloroplasts, and plant cells.^26^

One open area for advancing our understanding of dynamic photooxidative chemistry occurring in the photosynthetic apparatus is the quantitative detection of ^1^O_2_* and changes in its steady state concentration during illumination. Deciphering the participation of ^1^O_2_* in *in situ* functioning of photosynthetic light harvesting regulation is a longstanding challenge in the field. In principle, detection of luminescence (^1^Δ_g_ → ^3^∑_g_^–^) provides direct evidence for the existence of the ^1^O_2_* excited state. However, ^1^O_2_* emission occurs in the near-IR spectral region and its detection is complicated by spectral congestion, a short lifetime, and quenching in an aqueous solution.^27^ More commonly, exogenous fluorophores are utilized to selectively detect ^1^O_2_* in photosynthesis. However, the bulky and nonpolar molecular structures of abiotic fluorophores can lead to difficulties in their localization at the site of ^1^O_2_* generation. Commercially available probes, such as singlet oxygen sensor green (SOSG),^28^ are also known to undergo “self” photosensitization upon visible light illumination.^29−32^ To overcome these experimental difficulties, we set out to develop an alternative method for monitoring the concentration of ^1^O_2_* in intact photosynthetic systems and its temporal dynamics during illumination.

Electron paramagnetic resonance (EPR) spectroscopy is a well-established technique that is capable of detecting unpaired electrons with high sensitivity. The spin trap 2,2,6,6-tetramethylpiperidine (TEMP) reacts with ^1^O_2_* to form EPR-active nitroxide radical species 2,2,6,6-tetramethylpiperidine 1-oxyl (TEMPO).^33^ In 1994, Hideg *et al.* demonstrated spin-trapping EPR for probing the involvement of ^1^O_2_* during photoinhibition in plant thylakoid membrane suspensions.^34^ However, the connection between ^1^O_2_* photosensitization and NPQ and the temporal changes in the concentration of ^1^O_2_* were left uninvestigated. Here, we build on the method of Hideg *et al*. with the goal of tracking the real-time involvement of reactive ^1^O_2_* during photoprotection. In control experiments using dye-based systems, we defined the correlation of EPR spin probe signals to illumination conditions. We then extend the snapshot EPR methodology to thylakoid membranes for gaining insight into the ^1^O_2_* stress response during excess light exposure. Our results support the conventional model of NPQ in regulating the amount of ^1^O_2_* produced in the photosynthetic apparatus during short-term photooxidative stress.

## Materials and Methods

### Thylakoid Membrane Preparation

Preparation of thylakoid membranes was performed in a dark room under green light. Baby spinach leaves (280–450 g, store-bought) were washed and dark-adapted overnight at 4 °C. Thylakoid membranes were purified according to the protocol of Utschig *et al*.^35^ Destemmed leaves were blended (Sunbeam, 1.5 L) in 300–500 mL batches of ice-cold grinding buffer with 3–5 short pulses of 1 s duration, followed by filtration through a Hamilton Beach Big Mouth juice extractor. The grinding buffer (pH 7.5) contained 0.4 M NaCl, 20 mM HEPES, 4 mM MgCl_2_, 5 mM EDTA, and 1 mg/mL fatty acid-free BSA. The spinach solution was then transferred to centrifuge bottles and spun in a Beckman Coulter Avanti J-26 XP centrifuge set to 4 °C for 6 min at 6500*g*. The supernatant was discarded, and the pellet was gently resuspended in wash buffer (pH 6.5) containing 0.15 M NaCl, 20 mM HEPES, 4 mM MgCl_2_, 1 mM EDTA, and 1 mg/mL fatty acid-free BSA. Resuspended thylakoids were centrifuged for 1 min at 500*g*. The supernatant was collected and centrifuged for 6 min at 6000*g*. Pellets were resuspended using ∼30 mL suspension buffer and centrifuged for 8 min at 12,000*g*. The suspension buffer (pH 6.0) contained 15 mM NaCl, 20 mM HEPES, 5 mM MgCl_2_, 1 mM EDTA, 1 mg/mL fatty acid-free BSA, and 20% glycerol. Pellets were then resuspended in 30 mL suspension buffer and centrifuged for 8 min at 16,000*g*. Finally, pellets were resuspended in 6–8 mL suspension buffer and stored on ice until use.

The approximate chlorophyll concentration of each thylakoid preparation was measured in cold 80% acetone using a Beckman DU 800 spectrophotometer and quantified using the Arnon equations.^36^ The oxygen evolution activity of isolated thylakoids was confirmed using a Unisense Oxy-NP probe (Figure S1). Thylakoid samples were either used fresh by diluting the stock directly into buffer or frozen at −80 °C until use.

### EPR Spin-Trapping Measurements

Continuous-wave (cw) X-band (9.5 GHz) EPR spectra and time traces (kinetics) were measured using a Bruker ELEXSYS II E500 EPR spectrometer (Bruker Biospin Corp, Ettlingen, Germany) equipped with a TE_102_ rectangular EPR resonator (Bruker ER 4102ST). Samples were measured at room temperature in glass capillary tubes with 1 mm inner diameter. O_2_-saturated and O_2_-depleted samples were produced by bubbling the dye stock solutions and equilibrating the EPR capillary tube and syringe with a steady stream of oxygen or nitrogen gas, respectively. All EPR spectra were acquired using field modulation (100 kHz) with an amplitude modulation of 2 G and phase-sensitive lock-in detection, leading to first derivative-type spectra. Unless otherwise specified, spectra were acquired using 12.6 mW microwave power (12 dB attenuation).

To illuminate samples for photosensitization of singlet oxygen, a daylight white LED (SOLIS-3C, Thorlabs) was focused into the EPR cavity with illumination covering the entire visible spectral region from 400 to 800 nm (LED spectrum shown in Figure S2). The photosynthetic photon flux density (PPFD, units of μmol m^–2^ s^–1^) was adjusted by altering the brightness of the LED with approximate values specified in each figure caption. The spin traps (2,2,6,6-tetramethylpiperidine (TEMP) and 4-hydroxy-2,2,6,6-tetramethylpiperidine (TEMP-OH)) and spin label (TEMPO) were purchased from Sigma-Aldrich and used as received.

### EPR “Snapshot” Measurements of Singlet Oxygen in Thylakoid Membranes

Purified thylakoid membranes (∼2.8 mg Chl mL^–1^) were diluted to a final concentration of ∼250 μg Chl mL^–1^ in glycerol resuspension buffer (15 mM NaCl, 20 mM MES, and 20% glycerol in Milli-Q water at pH 6) in the presence of 0.5 mM ATP. Where specified, 2 μL of nigericin (50 mM stock prepared in ethanol) was also added at a final concentration of 100 μM. The thylakoid suspension was stirred and preilluminated with 1000 μmol m^–2^ s^–1^ of white light for a set duration. Following the preillumination period, 50 μL of TEMP (1 M stock prepared in acetonitrile) was added to reach a final concentration of 50 mM and incubated for 1 min with continued stirring. As a control experiment, in place of TEMP, 50 μL of the TEMPO nitroxide radical (600 μM stock prepared in acetonitrile) was added to reach a final concentration of 30 μM to assess the stability of TEMPO and any degradation processes leading to destruction of the EPR signal.

To avoid the HL-induced decrease in the TEMPO nitroxide radical signal that was observed during extended illumination of thylakoid membranes in the EPR cavity, the illumination period in the presence of TEMP was limited to 1 min, corresponding to the approximate time for generating a maximal TEMPO signal. Following 1 min illumination in the presence of TEMP, aliquots were transferred to a microcentrifuge tube and immediately flash-frozen with liquid nitrogen, a process that took 15–30 s in total. Frozen samples were stored in the dark at −80 °C until measurement. All EPR experiments were performed within 10 days of freezing the thylakoid sample.

Prior to EPR measurements, each sample was thawed and promptly transferred to an EPR capillary tube (1 mm inner diameter), a process that took 1–2 min in total. Care was taken to minimize illumination of the sample during this process. A cw X-band EPR spectrum was recorded for each sample at room temperature. Spin quantification was performed using the Xepr Spin Count function to quantify the concentration of TEMPO in each sample. This function relies on double integration of the first derivative-type cw EPR spectra and takes the quality factor of the resonator and instrument parameters into account. As a secondary quantification, the relative intensities of the low-field (≈3358 G) or midfield EPR peaks (≈3375 G) of the nitroxide were compared to estimate the changes in TEMPO signal, yielding similar results.

### Fluorescence Lifetime Measurements

Prior to florescence lifetime measurements, the thylakoid suspension was diluted in glycerol resuspension buffer (pH 6) with 50 μM methyl viologen as a secondary electron acceptor and 0.5 mM ATP to mediate ATP hydrolysis. Thylakoid membrane samples were placed in a 1 cm quartz cuvette with a microstir bar. To induce NPQ in the membranes, thylakoids were illuminated with 1000 μmol m^–2^ s^–1^ of white light provided by an LED (SOLIS-3C, Thorlabs). At specified time points, a 300 μL aliquot of the sample was transferred to a 1 mm cuvette (21-Q-1, Starna Cells) and measured by time-correlated single-photon counting, where the combined transfer and measurement process took ∼30 s.

Excitation was provided by a 405 nm laser diode (PC-405B, Picoquant) at a repetition rate of 20 MHz with a power of 360 μW at the sample. Chl *a* fluorescence emission was collected using a 40 mm focal length collection lens, directed to a monochromator (SP2300, Acton) set to 680 nm with a slit width of 25 μm, and detected using an avalanche photodiode (PDM, OptoElectronics Corp.). The photon arrival time histogram bin width was 25 ps, and emission was integrated for 4 s per snapshot. Fluorescence lifetimes were fit using an exponential decay function. The extent of quenching was calculated as , where τ_dark_ is the original fluorescence lifetime prior to illumination and τ(*t*) is the fluorescence lifetime at time point *t* during the 30 min HL illumination. Unlike fluorescence yield measurements, fluorescence lifetimes are not impacted by nonquenching processes, such as pigment bleaching. Under most experimental conditions, the long fluorescence lifetimes in the dark (>1 ns) indicate closure of PSII reaction centers (see discussion in refs (37) and (38)), eliminating contributions of photochemical quenching. As a control experiment, the addition of DCBQ, an artificial electron acceptor for PSII, resulted in a substantially shortened fluorescence lifetime due to increased activity of reaction centers, presumably because DCBQ replenishes the depleted plastoquinone pool (Figure S3).

## Results and Discussion

### EPR Spin-Trapping Method for Detection of Singlet Oxygen Photosensitization

**Figure 1:**
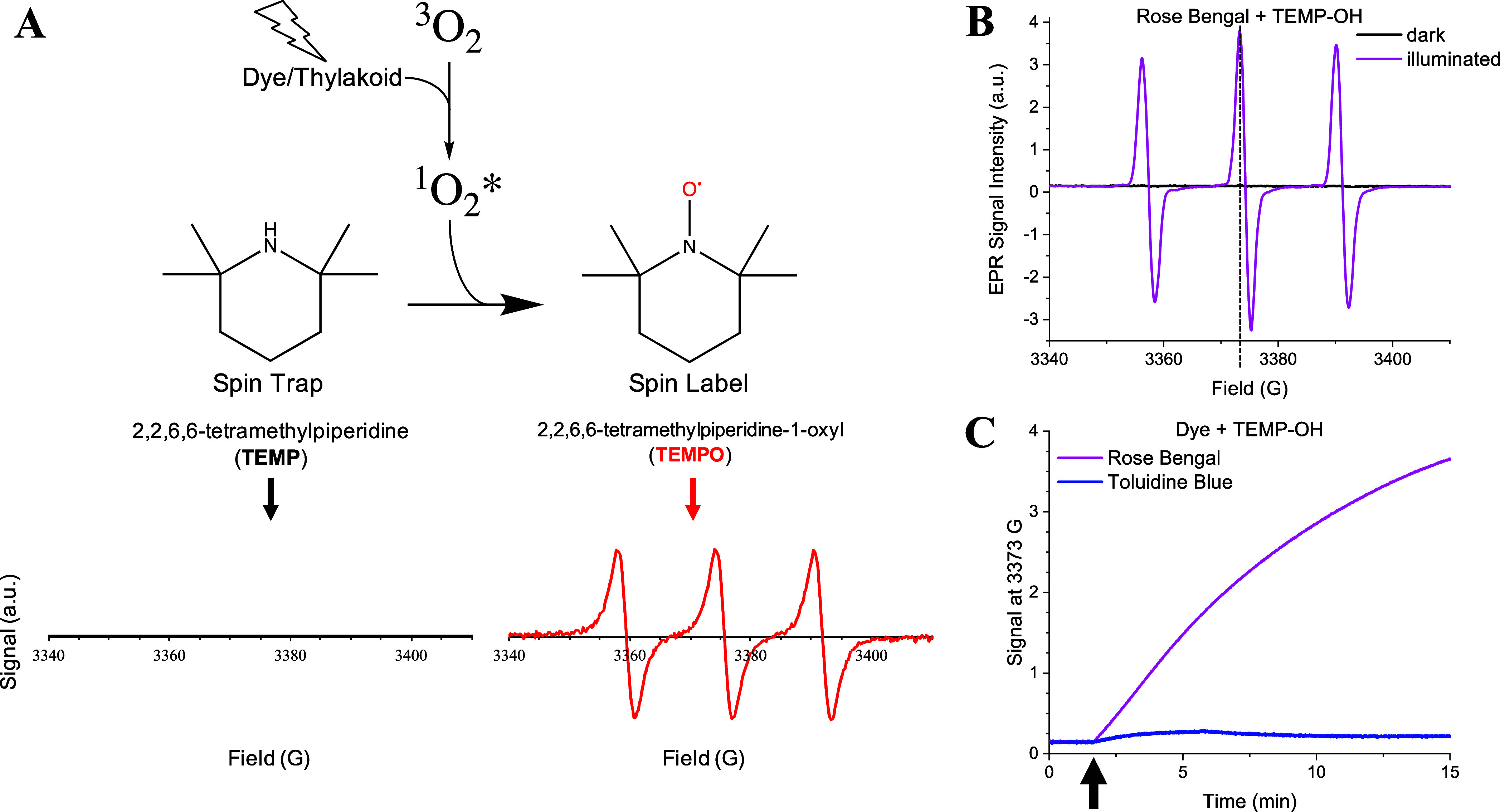
(A) Schematic for the detection of photosensitized ^1^O_2_* using TEMP as a spin trap. The reaction of ^1^O_2_* with the spin trap (TEMP, non-EPR-active) produces the EPR-active nitroxide radical TEMPO, detectable as a triplet in the EPR spectrum. For monitoring ^1^O_2_* in an aqueous solution, 4-hydroxy-2,2,6,6-tetramethylpiperidine (TEMP-OH) was employed in place of TEMP, which yields an identical EPR spectrum. (B) Spectra of RB + TEMP-OH measured before and after illumination. The vertical line depicts the midfield peak (3373 G) used for monitoring the kinetics of ^1^O_2_* photosensitization. (C) Time traces show comparison of toluidine blue (TB) and rose bengal (RB) as photosensitizers for production of ^1^O_2_*. The LED was turned ON at 2 min (see upright arrow) at a PPFD of ∼1780 μmol m^–2^ s^–1^. (A) 15 μM TEMPO in acetonitrile; (B, C) 0.2 mM RB or TB and 10 mM TEMP-OH. All EPR spectra and traces were measured at room temperature with 2G modulation amplitude and 12 dB attenuation.

We selected two commercially available dyes that are commonly used for photosensitization of ^1^O_2_*, toluidine blue (TB)^33,34,39^ and rose bengal (RB),^40−42^ to validate the EPR spin-trapping method for detection of ^1^O_2_* in a system significantly less complicated than that of the thylakoid membrane. Following excitation of the dyes to the S_1_ singlet excited states (UV–visible absorption spectra shown in Figure S4), a nanosecond-timescale intersystem crossing to the longer-lived triplet state (S_1_ → T_1_) can lead to triplet–triplet energy transfer in which the T_1_ state of the chromophore is quenched by the ground state of molecular oxygen. The ^1^O_2_* product of this photosensitization process can react with the spin trap TEMP, yielding the TEMPO nitroxide radical (Figure 1A), which can be detected by EPR. In lieu of the lipophilic TEMP, a more hydrophilic version of the spin trap (TEMP-OH with a hydroxyl group at the 4′ position) was used for detection of ^1^O_2_* in aqueous solutions.

As expected, upon illumination of an aqueous solution of RB and TEMP-OH in the EPR cavity with white light at an environmentally relevant PPFD level of 1000 μmol m^–2^ s^–1^, we observed an increase in the EPR signal, confirming the conversion of the EPR-silent spin trap to the EPR-active TEMPO nitroxide radical (Figure 1B). The rate of the signal increase was highly dependent on the light intensity: at low PPFD (∼400 μmol m^–2^ s^–1^), the signal continued to increase for at least 10 min, while at higher PPFD (∼4000 μmol m^–2^ s^–1^), the maximal signal was reached more quickly, even though the overall amount of signal was identical (Figure S5). In either case, prolonged illumination resulted in decreasing EPR signal with a rate that again depended on illumination intensity (higher light intensity resulted in faster decay). Illumination of an aqueous solution of RB and TEMP generated a much larger TEMPO signal compared to TB (Figure 1C). This difference can be explained by the higher quantum yield of intersystem crossing (Φ_ISC_) for RB relative to TB, likely enabled by the presence of multiple heavy atoms (halogen substituents) in RB.^42,43^ It is also consistent with a reported ∼75% singlet oxygen quantum yield (Φ_Δ_) for RB compared to ∼50% for TB at neutral pH.^39,44,45^ Given its high Φ_ISC_ and Φ_Δ_ leading to a significant TEMPO signal, RB was chosen as the optimal dye for systematic investigation of the effects of light intensity and dissolved O_2_ concentration on ^1^O_2_* photosensitization.

**Figure 2:**
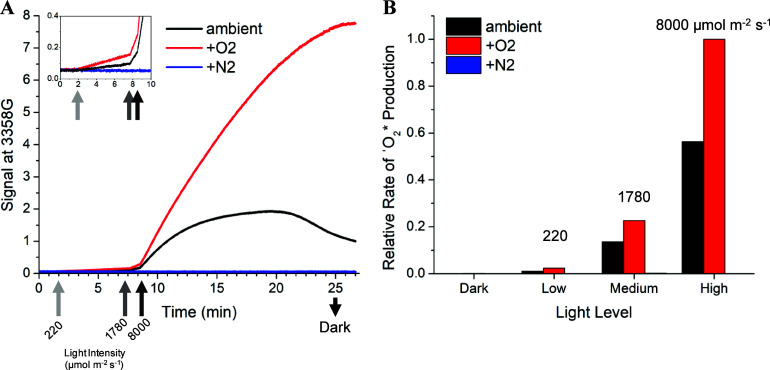
Kinetics of singlet oxygen photosensitization by RB (0.2 mM), detected by TEMP-OH (10 mM) in water. (A) Time traces for the EPR signal monitored at 3358 G for ambient, O_2_-saturated (+O_2_), and O_2_-depleted (+N_2_) samples with direct illumination in the EPR cavity. The LED intensity was increased at time points denoted by upward-facing arrows, and the LED was switched off at 25 min. Inset: enlargement of the first 10 min. (B) Relative rates of singlet oxygen photosensitization for each illumination intensity, taken as the initial slope of the EPR signal increase at 3358 G during the first 100 s of each illumination condition. Dark, low, medium, and high light levels correspond to 0, 220, 1780, and 8000 μmol m^–2^ s^–1^, respectively.

In addition, we illuminated an aqueous solution of 0.2 mM RB and 10 mM TEMP-OH directly in the EPR cavity. Upon exposure to low PPFD (220 μmol m^–2^ s^–1^), we observed an increase in EPR signal at 3358 G corresponding to the TEMPO product (Figure 2). Upon increasing the PPFD to 1780 μmol m^–2^ s^–1^, the slope of the signal increased, and a subsequent increase in the PPFD to greater than 8000 μmol m^–2^ s^–1^ resulted in an even larger increase in signal. Compared to an ambient solution, the oxygen-saturated solution of RB and TEMP-OH showed much larger increases in signal at all three PPFDs, likely due to the availability of more O_2_ to react with the triplet excited state of RB. Conversely, as a control experiment, an oxygen-depleted solution of RB and TEMP-OH showed no increase in signal due to the absence of O_2_ precluding energy transfer between RB and molecular O_2_.

To conclude, RB is an excellent photosensitizer for production of ^1^O_2_* in an aqueous solution, resulting in a roughly ∼17-fold higher EPR signal than TB under similar conditions (Figure 1C). Both dyes exhibit photosensitization of ^1^O_2_* with rates of TEMPO generation that scale with increasing PPFD. As expected, the rate of signal increase depends on the dissolved O_2_ content, with larger TEMPO signals being observed for an O_2_-saturated solution. Longer illumination eventually results in destruction of the nitroxide radical TEMPO signal, a process that is also dependent on the PPFD.

### EPR Detection of Singlet Oxygen Production in Thylakoids during Illumination

**Figure 3:**
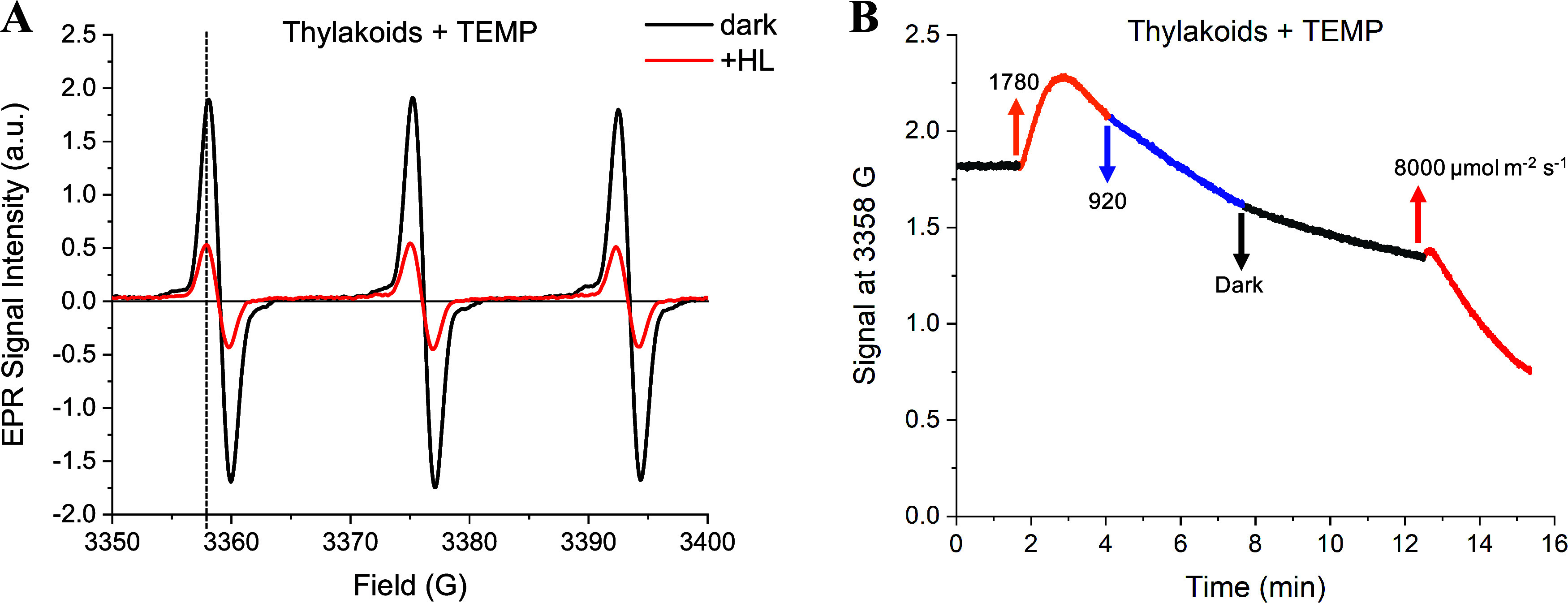
EPR spectral and kinetic analysis of ^1^O_2_* in purified thylakoid membranes. (A) EPR spectra before illumination in the dark (black) and after prolonged illumination (red). The spin trap TEMP (2,2,6,6-tetramethypiperidine) was used at a concentration of 50 mM in glycerol resuspension buffer (pH 6) with 0.5 mM ATP. The dashed vertical line corresponds to the low-field peak (3358 G) used for tracking illumination-induced kinetics. (B) Time trace of the EPR signal at 3358 G for the same thylakoid membrane sample exposed to changes in illumination intensity inside the EPR cavity. Upward- and downward-facing arrows indicate the timing for light intensity changes (values specify PPFD in μmol photons m^–2^ s^–1^). The thylakoid concentration was approximately 500 μg Chl/mL. Additional thylakoid membrane aliquots exposed to different light sequences are shown in Figure S6.

**Figure 4:**
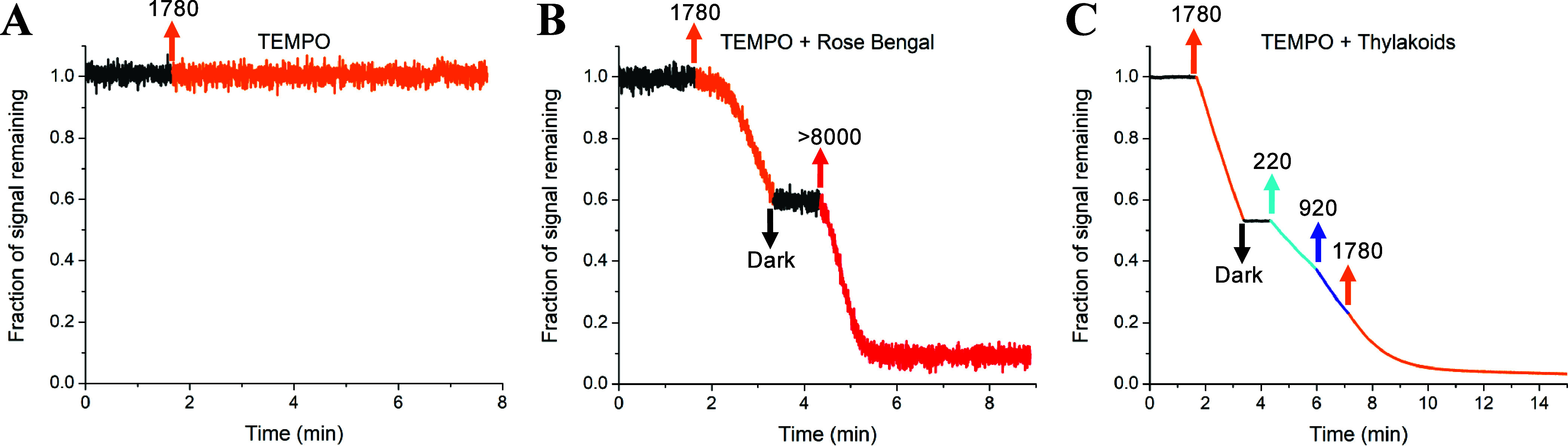
Light-intensity dependence of the nitroxide radical concentration. Time traces for the EPR signal at 3358 G for (A) 15 μM TEMPO in acetonitrile, (B) 15 μM TEMPO in the presence of a photosensitizer (RB) in acetonitrile, and (C) 300 μM TEMPO in a thylakoid membrane suspension at 500 μg Chl/mL with 0.5 mM ATP dissolved in glycerol resuspension buffer (pH 6). There was no TEMP present; hence, no increase in EPR signal is expected and each trace is instead plotted as the fraction of the original signal remaining. The starting signal intensity at 3358 G was ∼0.3 for 15 μM TEMPO in acetonitrile (A, B) and ∼14.5 for 300 μM in thylakoid suspension (C). Upward- and downward-facing arrows indicate the timing for light intensity changes where labels specify the approximate light intensity (μmol photons m^–2^ s^–1^).

To assess the effect of thylakoid membrane illumination on the detectable EPR signal of TEMPO, we incubated thylakoids with TEMP and recorded the X-band EPR spectrum in the dark and after illumination. The initial TEMPO signal recorded before illumination (Figure 3A) represents typical impurity in the TEMP spin trap on the order 0.1%. A fraction of this species may also be generated by redox reactions under ambient conditions in a complex redox-active biological system like thylakoid membranes. Upon light illumination of the TEMP-containing thylakoids, we observed complicated kinetics of the TEMPO EPR signal, which depend upon light intensity and duration of the illumination (Figure 3B). It is important to note that the initial growth of the TEMPO EPR signal is due to the reaction of ^1^O_2_* with the spin trap TEMP. Control experiments discussed below and shown in Figure 4 confirm that illumination of TEMPO alone cannot lead to an increase in the TEMPO EPR signal but only to a decrease in the signal.

The light dependence of the EPR signal of the nitroxide TEMPO (Figure 3B) demonstrates that TEMP is capable of monitoring ^1^O_2_* in the thylakoid membrane. The observed decay of the TEMPO signal during prolonged illumination is likely due to a combination of ^1^O_2_* and the highly redox active environment of the thylakoid membrane, which has previously been shown to cause reduction of TEMPO, leading to a loss of EPR signal.^46^ Kinetic analysis of the low-field EPR peak at 3358 G showed that the magnitude of the light-induced decrease in signal scaled with the PPFD (Figure S6). ATP was included in the thylakoids to mediate ATP hydrolysis.^47^ Control experiments show no effect of ATP on nitroxide EPR signals (Figure S7).

To gain further insight into the origin of the decreasing TEMPO signal during illumination of thylakoid membranes, the intensity of the low-field peak at 3358 G (Figure 3A) was tracked for several samples in the presence of high concentrations of the TEMPO nitroxide radical. A sample of 15 μM TEMPO without dye in acetonitrile showed no change in TEMPO signal (Figure 4A), indicating that the nitroxide radical is stable during illumination. In contrast, 15 μM TEMPO in the presence of 0.1 mM RB showed a sizable decrease in signal during illumination (Figure 4B), demonstrating the effect of ^1^O_2_* on the decay of TEMPO. Likewise, thylakoid membranes incubated with 300 μM TEMPO showed a very large decrease in signal during illumination, with the signal entirely consumed within 15 min of illumination at PPFDs less than 2000 μmol m^–2^ s^–1^ (Figure 4C). Therefore, it was concluded that illumination of the TEMPO nitroxide radical itself is not directly responsible for the decrease in EPR signal. Instead, such a decrease only occurs in the presence of a light-absorbing molecule, either dye or Chl, and the rate of the decrease is faster for higher PPFD. This suggests that an interaction between the excited states of the photosensitizer molecules and TEMPO may occur during continuous illumination. Additionally, chemical reactions leading to TEMPO degradation, such as those documented for TEMPO derivatives exposed to hydroxyl radicals,^48^ might contribute to the observed decrease in EPR signal during prolonged illumination.

The possibility of a secondary light-dependent reaction resulting in reduction of EPR-active TEMPO to an EPR-silent hydroxylamine implies that EPR detection of ^1^O_2_* is an underestimate of the real ^1^O_2_* concentration.^49^ Such a reaction resulting in the disappearance of EPR signal during illumination has been observed in isolated LHCII proteins, with a rate that depended on the PPFD.^9^ Therefore, side reactions of TEMPO pose a challenge for EPR-based detection of ^1^O_2_* in plant systems due to the high concentration of redox-active equivalents generated during illumination of thylakoids. One possible solution is to attempt to reoxidize the diamagnetic TEMPO species back to the radical form via aeration in the presence of lead oxide, followed by extraction into ethyl acetate.^9,34^ Separately, a decrease in the TEMPO EPR signal has been previously observed in thylakoid membrane systems, which was suggested to be due to the formation of a nonbilayer phase of the thylakoid membrane lipids.^50^ It has also been suggested that a temporary burst of singlet oxygen produced by free and solubilized Chl may occur at the beginning of illumination,^8^ although the mechanism remains undetermined.

### EPR “Snapshot” Spectroscopic Studies of Photoprotection in Thylakoids

To track the relative levels of ^1^O_2_* in thylakoid membranes during photoprotection, we took advantage of the time interval associated with the maximal TEMPO signal during HL (see Figure 3B). By limiting the illumination of the thylakoids in the presence of TEMP to exactly 1 min, we hypothesized that we could minimize the decrease in TEMPO signal that occurs during extended periods of illumination and gain insights into the temporal dynamics of ^1^O_2_* production and its regulation in thylakoid membranes. Following 1 min of illumination at 1000 μmol photons m^–2^ s^–1^, the TEMPO concentration in a representative thylakoid aliquot increased by 46% as a result of production of ^1^O_2_* and its subsequent trapping by TEMP (Figure S8). The increase in TEMPO signal indicates that a 1 min illumination of the thylakoids in the presence of TEMP followed by EPR measurement is well suited for detecting ^1^O_2_* produced by HL-exposed thylakoid membranes, consistent with the EPR time traces shown in Figure 3B. We confirmed that the spin concentration remained identical when reacquiring a spectrum for the same sample after rapid freezing and thawing of the capillary, indicating that the process of freezing and thawing a preilluminated thylakoid sample does not alter the measurable concentration of TEMPO (Figure S8).

**Figure 5:**
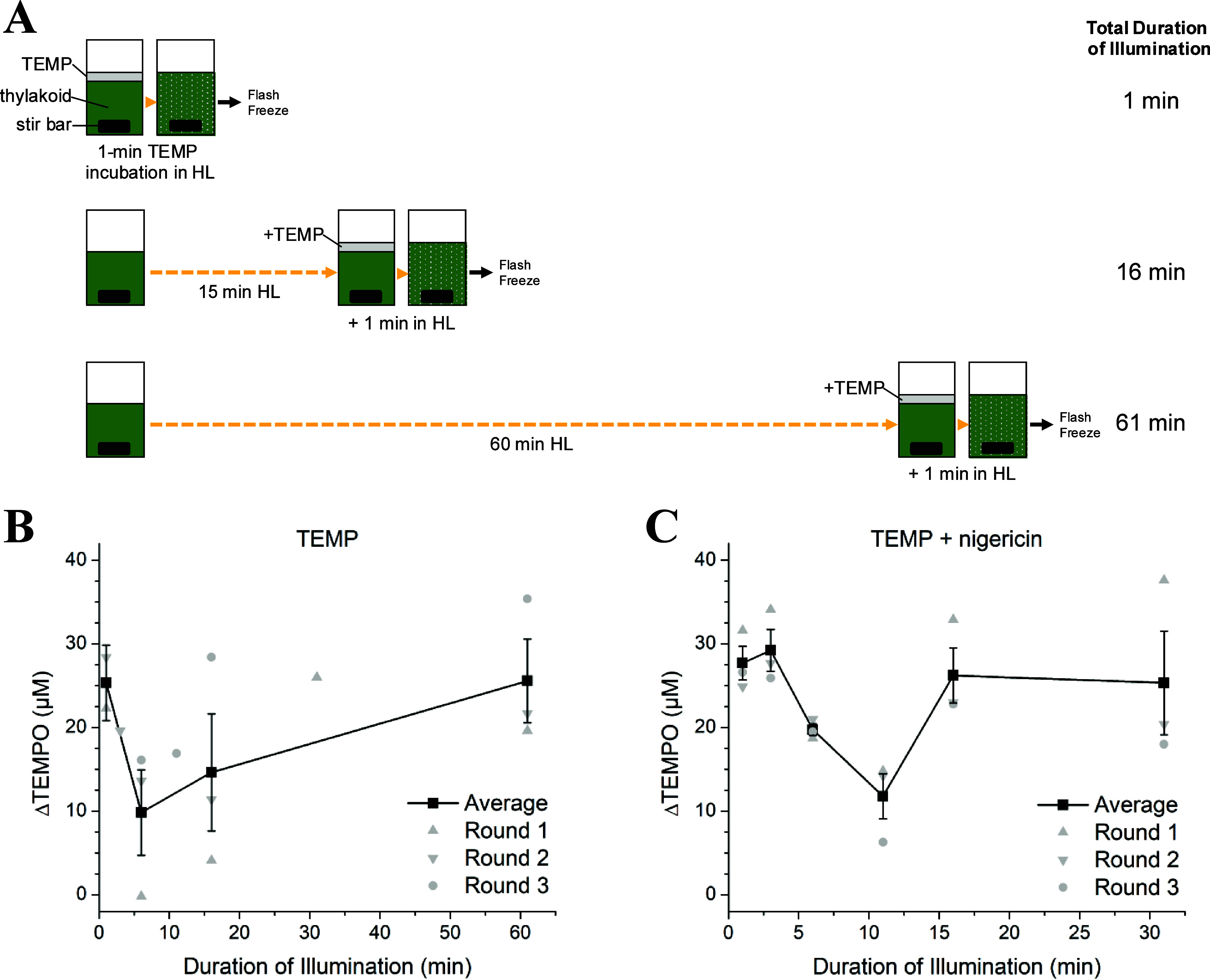
“Snapshot” EPR experiment method for monitoring ^1^O_2_* in thylakoid membranes (∼250 μg Chl mL^–1^) during HL exposure. (A) Schematic showing the experimental design for assessing light-induced changes in ^1^O_2_* concentration in thylakoid membranes. Thylakoid aliquots were illuminated at 1000 μmol photons m^–2^ s^–1^ in separate cuvettes with stirring. The spin trap TEMP was added to each cuvette at a concentration of 50 mM for the final minute of illumination. After the 1 min HL incubation period, the sample was immediately flash-frozen, a process that took 15–30 s. Thylakoid aliquots were stored in the dark at −80 °C and used within 10 days. For measurement, samples were thawed and immediately transferred to an EPR capillary tube. (B) EPR snapshot measurements of ^1^O_2_* formed in untreated thylakoid membranes or (C) membranes treated with 100 μM nigericin as a function of the total duration of HL treatment. Individual replicates are presented as gray markers, while the average of replicates and standard error of mean (*n* = 3) are indicated in black. The TEMPO concentration in each thylakoid aliquot was quantified by double integration of each EPR spectrum. The concentration of TEMPO formed in each HL-exposed thylakoid aliquot (ΔTEMPO) was estimated by subtracting the baseline TEMPO concentration measured in control thylakoid samples that were incubated with TEMP for 1 min in the dark (TEMPO concentration: 34 ± 4 μM, *n* = 4 replicates). The uncorrected data are shown in Figure S9 along with snapshots for 1 min incubations with TEMPO.

We thus designed an experimental protocol for reliable detection of ^1^O_2_*: (1) expose thylakoid membranes to HL for a set length of time, (2) add TEMP and perform the final minute of HL to capture the ^1^O_2_* generated during the HL time period, and (3) freeze-quench the sample for later EPR analysis (see Figure 5A for the experimental design). We confirmed that rapid freeze quenching of the sample in the dark terminates side reactions involved in TEMPO decay, preserving the EPR-active product. Each thylakoid aliquot thus provides a “snapshot” for the amount of ^1^O_2_* produced by the membrane at various time points during a total duration of 1 h of HL exposure. We note that a similar experimental approach of storing preilluminated membrane samples in liquid nitrogen until measurement of the EPR spectrum at room temperature has been successfully employed for PSII-containing membranes.^46,51^ Measurement of ^1^O_2_* produced by thylakoid membranes has also been demonstrated using LC/MS-based detection of TEMPO, yielding comparable TEMPO concentrations to the EPR method.^52^

Using our “snapshot” EPR approach, we successfully observed changes in the apparent concentration of ^1^O_2_* in thylakoid membrane samples during HL exposure (Figure 5B,C). A significant decrease in the concentration of TEMPO occurred during the initial stage of illumination, with the lowest concentrations observed near the 5 min time point of illumination (Figure 5B). This represents an ∼65% decrease in the rate of ^1^O_2_* production by the thylakoid membrane during the first few minutes of HL exposure. Following the initial decrease, the concentration of TEMPO gradually increased, eventually returning to the initial value at 60 min of illumination.

We further investigated how the transmembrane proton gradient alters the dynamics of ^1^O_2_* in the thylakoid membrane. Treatment with the chemical uncoupler nigericin dissipates the transthylakoid pH gradient,^53^ preventing the conversion of violaxanthin to zeaxanthin and hindering the activation of NPQ. Like the untreated sample, thylakoids treated with nigericin showed a sizable decrease in TEMPO during the initial illumination period (Figure 5C). However, this decrease in signal was delayed by ∼5 min relative to the untreated sample, with the minimum TEMPO signal in the presence of nigericin observed around 10 min of illumination. Similarly, the timescale associated with the decrease in TEMPO signal was faster in the absence of nigericin (Figure S10), suggesting a possible dependence of ^1^O_2_* kinetics on ΔpH.

**Figure 6:**
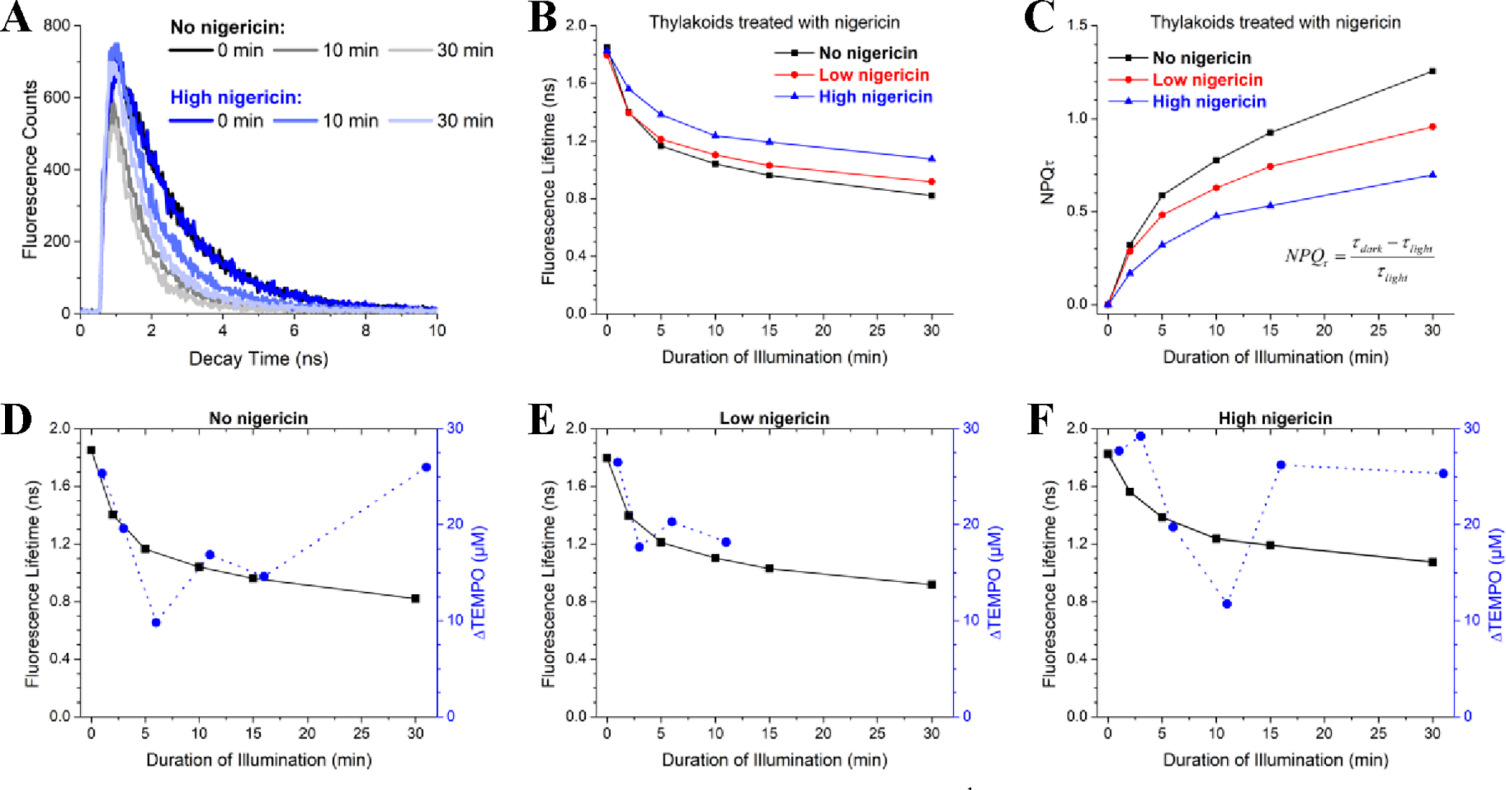
Correlation of nonphotochemical quenching kinetics and apparent ^1^O_2_* concentration in thylakoid membranes treated with various concentrations of nigericin. (A) Fluorescence decay curves for untreated and nigericin-treated thylakoids corresponding to 0, 10, and 30 min of illumination. (B) Average fluorescence lifetime and (C) calculated NPQ_τ_ values for each duration of illumination. (D–F) Comparison of fluorescence lifetime (black) and TEMPO concentration (blue) at specified intervals of illumination at 1000 μmol m^–2^ s^–1^. Untreated thylakoids (D) and thylakoids in the presence of 1 μM (E) or 100 μM (F) nigericin. Fluorescence lifetimes were measured in glycerol resuspension buffer (pH 6) with 50 μM methyl viologen and 0.5 mM ATP. TEMPO concentrations were measured as specified in Figure 5 and in Materials and Methods.

To elucidate the connection between ^1^O_2_* production and NPQ under HL, we compared our snapshot EPR measurements of TEMPO production with Chl fluorescence lifetimes measured on thylakoid samples under similar conditions (Figure 6A–C). Both the untreated and nigericin-treated thylakoid samples exhibited similar unquenched lifetimes prior to illumination (∼1.8 ns). The laser power, integration time, and long fluorescence lifetimes indicate successful closure of PSII reaction centers and minimal contributions of photochemical quenching in the measurements. Upon high-light exposure, the untreated sample showed a rapid quenching response, reaching a lifetime of ∼1.2 ns within 5 min of illumination due to NPQ. In contrast, the nigericin-treated sample showed less overall quenching (Figure 6C) and a delayed onset of NPQ, requiring >10 min of illumination to reach the same 1.2 ns lifetime (Figure 6B), likely due to a slower formation of ΔpH in the light.

The comparison of the EPR and time-resolved fluorescence spectroscopy data suggests two distinct stages in the production of ^1^O_2_* in thylakoid membranes. During the first stage, representing the membrane’s short-term response to HL, defined here as the first 10 min of illumination, the TEMPO signal is correlated to the average Chl fluorescence lifetime. As NPQ processes switch on, the shortening of the ^1^Chl* excited state lifetime leads to less ^1^O_2_* production (Figure 6D), detectable as decreased levels of TEMPO. The correlation between the trends in TEMPO concentration and dynamics of Chl fluorescence quenching is further supported by the nigericin-treated thylakoid sample, which exhibited an ∼5 min delay to reach the minimum TEMPO concentration (Figure 6F) alongside slower activation of NPQ.

Although the observed trends in TEMPO concentration correlated well with the dynamics of Chl fluorescence quenching for short illumination periods (Figure 6D–F), differences become apparent upon prolonged exposure to HL. After 15 min of illumination, the thylakoid samples show only relatively small changes in fluorescence lifetime due to nearly complete saturation of NPQ of the isolated membranes. Yet, a marked increase in TEMPO signal is observed, with the relative TEMPO concentration eventually matching the starting concentration (i.e., prior to the induction of any NPQ) following 30 min of HL (Figure 6D). Thus, increased amounts of ^1^O_2_* are produced upon saturation of all NPQ processes in thylakoid membranes.

**Figure 7:**
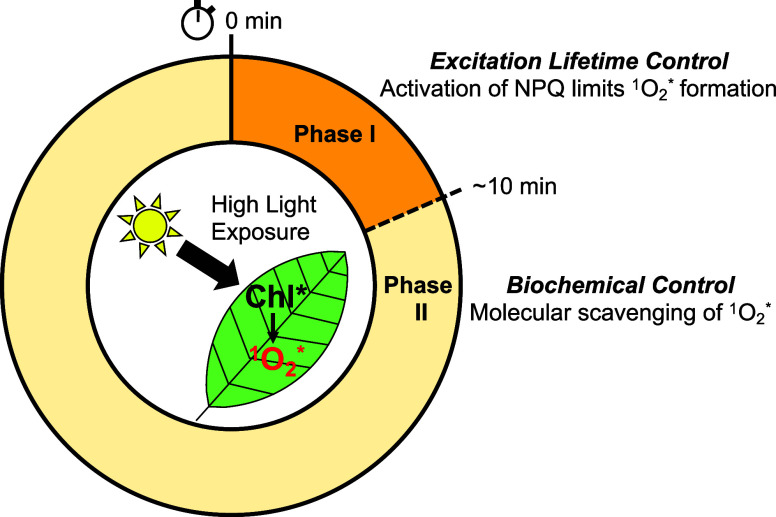
Proposed biphasic model for ^1^O_2_* production in photosynthetic systems during HL stress. Upon HL exposure, the activation of NPQ is one of the initial processes limiting the formation of ^1^O_2_*. The decreased Chl excited lifetime results in less ^1^O_2_* and therefore decreases the TEMPO signal during the first ∼10 min of illumination of the thylakoid membranes (phase I). On a longer timescale, biochemical regulatory processes, including scavenging of ^1^O_2_* by antioxidant molecules (carotenoids, lipids, proteins, etc.) combined with various signaling cascades in the cell, continue to modulate ^1^O_2_* levels. In our measurements of isolated thylakoid membranes, continued illumination saturates the antioxidant capacity, leading to increased production of ^1^O_2_* during phase II of HL exposure.

We propose that the biphasic temporal trends in ^1^O_2_* can be understood in terms of two distinct mechanisms involved in regulation of ROS during HL stress (Figure 7). In the initial regime, the activation of NPQ limits the amount of ^1^O_2_* due to a decrease in the average lifetime of the Chl excited state, a response that is the strongest during initial exposure to HL. The correlation between NPQ activation and the decreased production of ^1^O_2_* supports the canonical view that the rapid activation of NPQ-related energy dissipation provides crucial regulation during short-term light stress^21,23^ by limiting the production of ^1^O_2_* and thus protecting the photosynthetic apparatus from ROS-induced photooxidative stress. Other than NPQ, on longer timescales, ^1^O_2_* levels are controlled by biochemical sinks for ROS removal via antioxidants. These sinks, including carotenoids,^54,55^ lipids,^13^ and proteins,^10^ are generally lipophilic molecules that are capable of chemical and physical scavenging of ^1^O_2_* molecules.^22,56^ The combined interaction of ROS and antioxidants in the photosynthetic apparatus creates a delicate balance of redox active species, which may participate (or provide input) into signaling cascades that can affect the system-wide plant function through gene regulation.^57,58^ We speculate that saturation of the antioxidant capacity for scavenging and removal of ^1^O_2_*—combined with the inability of isolated membranes to activate longer-term processes, such as modulation of gene expression—underlies the observed increase in ^1^O_2_* during phase II of the thylakoid’s HL response (Figure 7).

The precise cause of the increased ^1^O_2_* levels during prolonged HL exposure of thylakoid membranes awaits further investigation. In a fully intact photosynthetic system, longer-term regulatory mechanisms (sustained quenching, photoinhibition, PSII repair, etc.)^22^ together with stress signaling processes^25,59^ control *in vivo* ROS levels, enabling plant survival during extended periods of abiotic stress. The ongoing development of techniques for sensitive and specific measurements of ROS and other redox-active species will reveal new insights into the complex photooxidative chemistry occurring in the photosynthetic apparatus on timescales relevant to environmental perturbations.

## Conclusions

Singlet oxygen, an excited state of molecular oxygen, is an unavoidable byproduct of photosynthetic light-harvesting especially under HL conditions. While it has crucial roles in both damage and signaling, the temporal evolution of ^1^O_2_* in the photosynthetic apparatus during HL stress is largely unknown. In benchmark studies combining spin-trapping EPR and time-resolved fluorescence spectroscopies, we reveal the complex temporal dynamics of ^1^O_2_* during the first hour of HL illumination of spinach thylakoid membranes. We observe an interference effect on the nitroxide radical EPR signal that arises from direct illumination of chromophores, both in dye-based and natural Chl-based systems, which implies an underestimation of ^1^O_2_* levels by the spin trapping method. Nonetheless, we successfully demonstrate that the apparent ^1^O_2_* concentration in thylakoid membranes changes throughout different stages of HL illumination and offer new insights into the complicated photooxidative chemistry occurring in the photosynthetic apparatus. During the first regime, corresponding to the membrane’s initial response to HL, the relative concentration of ^1^O_2_* decreases concurrently with quenching of the Chl fluorescence lifetime. Afterward, upon saturation of NPQ, the concentration of ^1^O_2_* rises, reflecting the utility of NPQ in mitigating photooxidative stress in the short term. These findings support previous hypotheses that the physiological role of photoprotective quenching is to reduce the amount of singlet oxygen produced in the photosynthetic apparatus.

## Supporting Information Available

O_2_ evolution characterization of thylakoids (Figure S1), spectrum of LED light source (Figure S2), fluorescence lifetime measurements of thylakoids with MV and DCBQ (Figure S3), absorption spectra of photosensitizers (TB and RB) (Figure S4), light intensity dependence of ^1^O_2_* photosensitization by TB (Figure S5), additional replicates for the kinetics of ^1^O_2_* in thylakoids (Figure S6), EPR control measurements with ATP (Figure S7), freeze–thaw effect on the TEMPO EPR signal (Figure S8), spin concentrations measured in snapshot EPR spectroscopy during illumination of thylakoid membranes (Figure S9), and kinetic analysis of changes in TEMPO concentration during illumination (Figure S10) (PDF)
