# Supplementary material for: EPR Spin-Trapping for Monitoring Temporal Dynamics of Singlet Oxygen during Photoprotection in Photosynthesis
Source: Biochemistry. 2024 Apr 29;63(9):1214–24. doi: 10.1021/acs.biochem.4c00028 (PMC11080054; doi:10.1021/acs.biochem.4c00028)
Supplement: Supplementary file 1 — bi4c00028_si_001.pdf [file bi4c00028_si_001.pdf]

*Supporting Information:*

# EPR Spin-Trapping for Monitoring Temporal Dynamics of Singlet Oxygen during Photoprotection in Photosynthesis

Collin J. Steen,[1,2,*] Jens Niklas,[3] Oleg G. Poluektov,[3] Richard D. Schaller,[4] Graham R. Fleming,[1,2] Lisa M. Utschig[3]

1 Department of Chemistry, University of California, Berkeley, CA, 94720

2 Molecular Biophysics and Integrated Bioimaging Division, Lawrence Berkeley National Laboratory, Berkeley, CA, 94720

3 Chemical Sciences and Engineering Division, Argonne National Laboratory, Lemont, IL 60439

4 Center for Nanoscale Materials, Argonne National Laboratory, Lemont, IL 60439

*Corresponding Author: collin.steen@berkeley.edu

# Contents

**Figure S1**: Characterization of $O_2$ evolution capability of thylakoids.

**Figure S2**: Spectrum of light source.

**Figure S3**: Fluorescence lifetime measurements of thylakoids with MV and DCBQ.

**Figure S4**: Absorption spectra of photosensitizers (TB and RB).

**Figure S5**: Light intensity dependence of $^1O_2^*$ photosensitization by TB.

**Figure S6**: Additional replicates for the kinetics of $^1O_2^*$ in thylakoids.

**Figure S7**: EPR control measurements with ATP.

**Figure S8**: Freeze-thaw effect on TEMPO EPR signal.

**Figure S9**: Spin concentrations measured in snapshot EPR spectroscopy during illumination of thylakoid membranes.

**Figure S10**: Kinetic analysis of changes in TEMPO concentration during illumination.

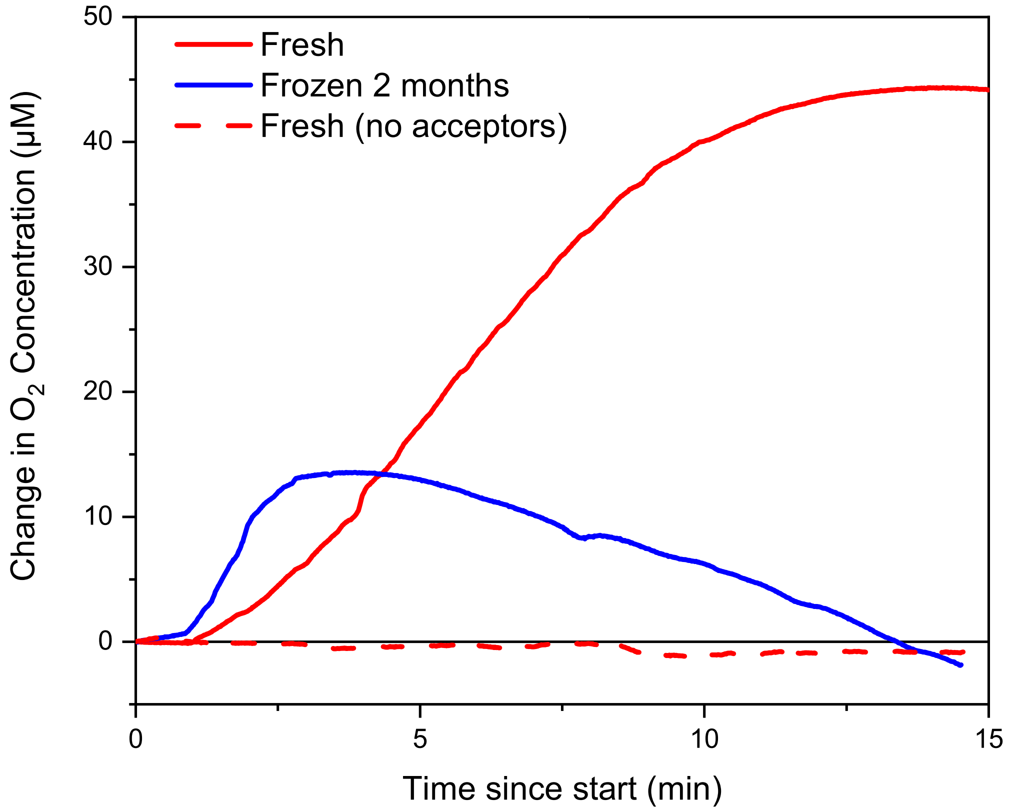


**Figure S1**. Characterization of oxygen evolution capabilities of thylakoid membrane preparation showing a comparison of fresh thylakoids (1 day old) and 2-month-old frozen thylakoids in the presence and absence of electron accepting molecules (250 μM DCBQ and 1 mM ferricyanide) in glycerol resuspension buffer (pH 6). All O2 evolution curves were measured using a Unisense Oxy-NP probe with a 2-point $O_2$ concentration calibration, ranging from 0 to 278 μM. Prior to measurement, the buffer was bubbled with $N_2$ for at least 30 min to minimize the concentration of $O_2$. Immediately prior to measurement, thylakoid stock was diluted into buffer at a concentration of 80 μg Chl/mL in a septa-capped cuvette equipped with a stir bar and stored in the dark. For each experiment, the LED was turned ON at 1 min.

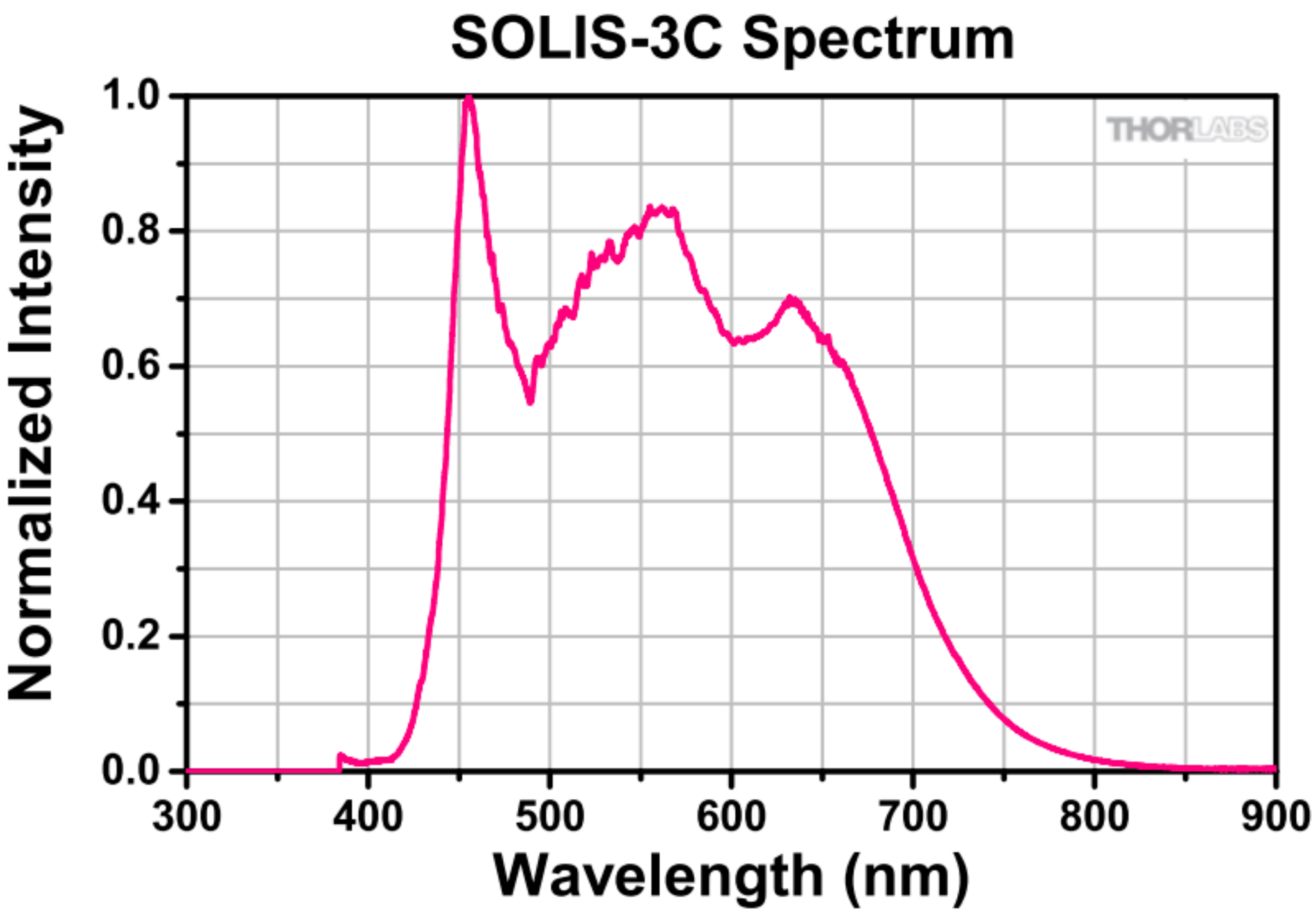


**Figure S2**. Spectrum of the daylight white LED (SOLIS-3C, ThorLabs) used as a light source in this study.

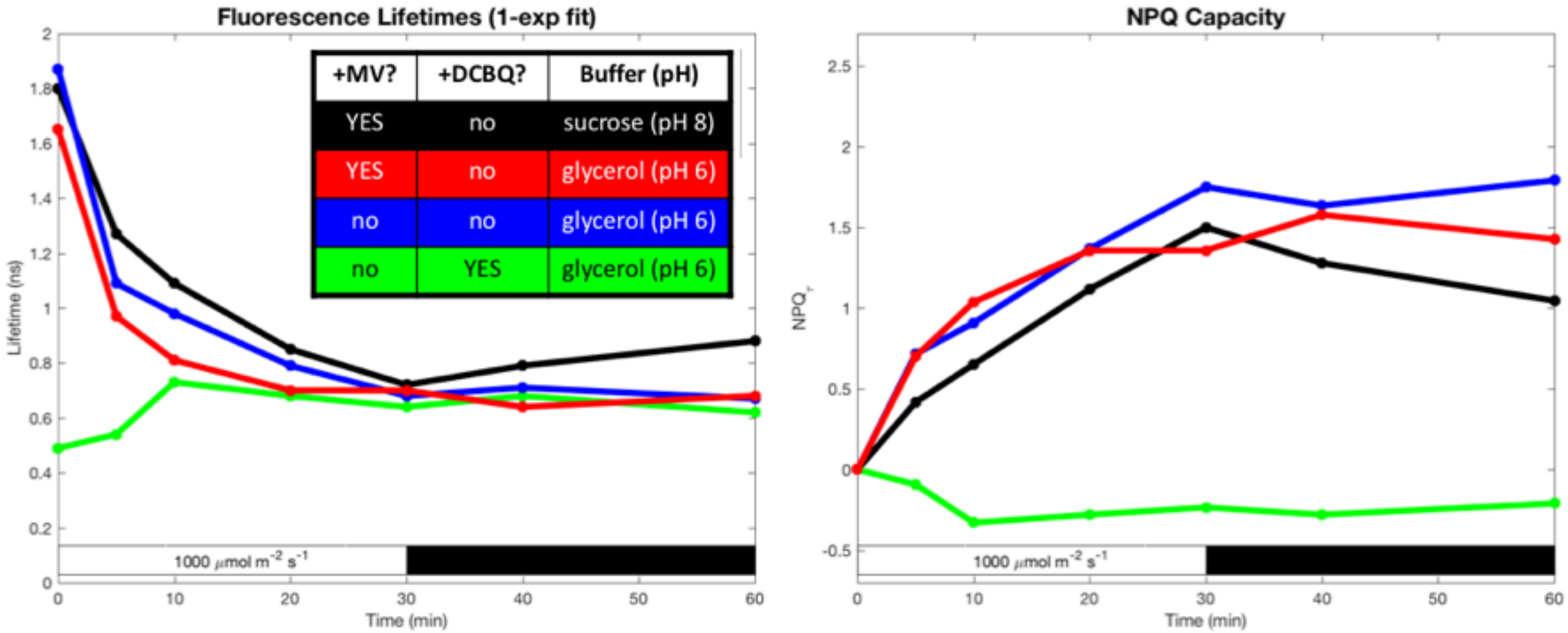


**Figure S3.** Effect of methyl viologen (MV, 50 uM), DCBQ (DCBQ, 250 uM), and buffer pH on fluorescence lifetime measurements of NPQ. Similar NPQ magnitude and kinetics were observed in the presence and absence of MV, independent of buffer composition and pH. Inclusion of DCBQ, an artificial electron acceptor for PSII, resulted in significantly shorter lifetimes presumably because DCBQ replenishes the depleted plastoquinone pool leading to higher activity of PSII reaction centers and associated photochemical quenching of Chl fluorescence.

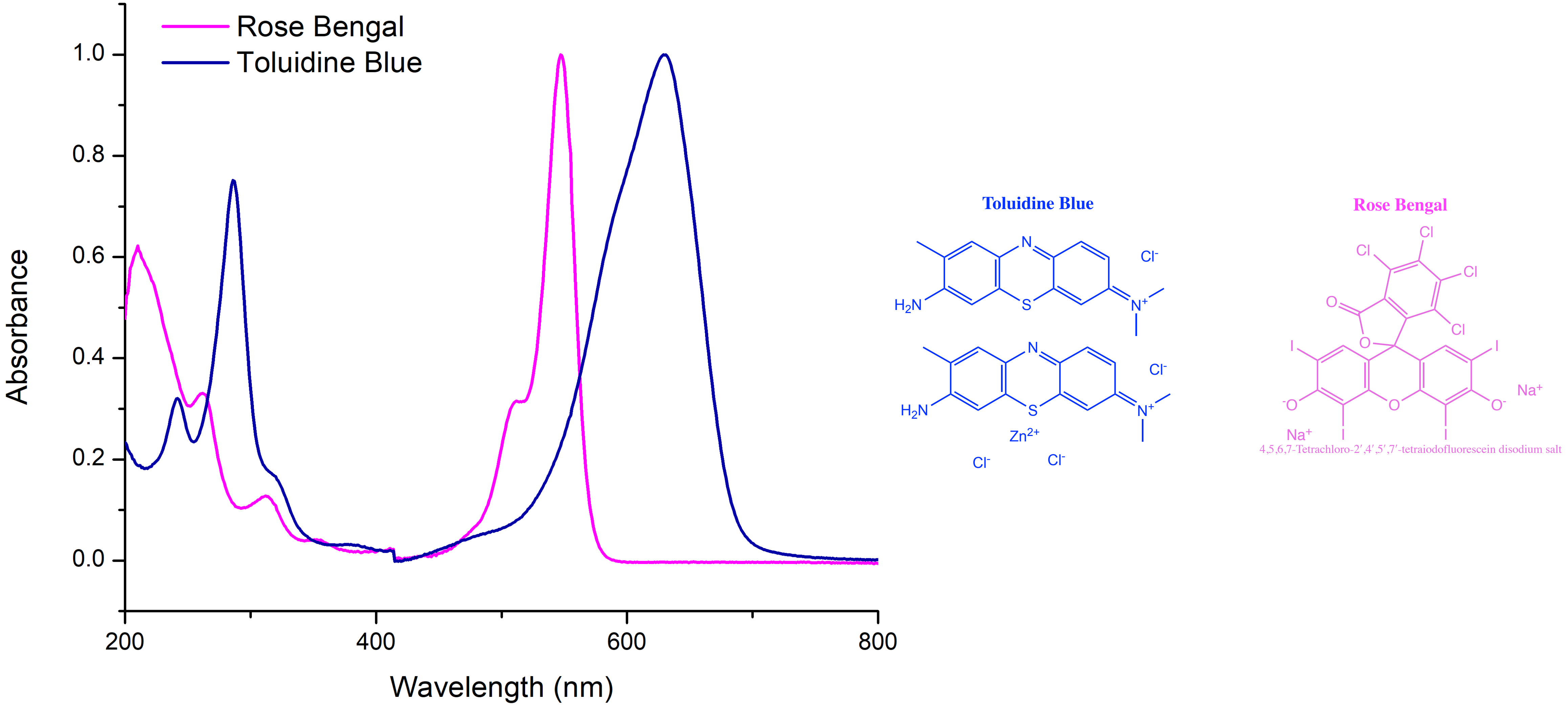


**Figure S4.** Absorption spectra and chemical structures for toluidine blue (TB, blue curve) and rose bengal (RB, pink curve) photosensitizers dissolved in milliQ water. Each spectrum is normalized at its maximum, 547 nm and 631 nm, respectively.

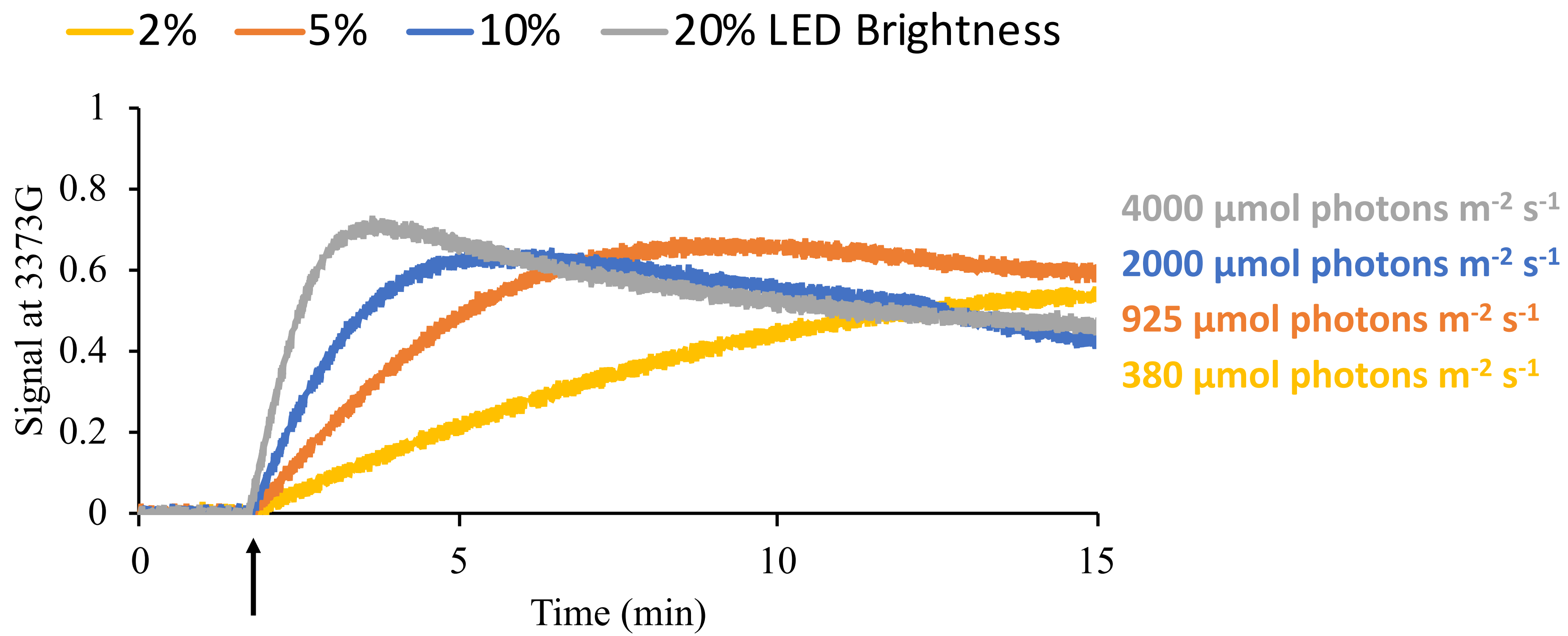


**Figure S5**. Photosensitized production of singlet oxygen in water. $^1O_2$* was produced by illumination of toluidine blue (TB) and was detected via 4-hydroxy-2,2,6,6-tetramethypiperidine (TEMP-OH). Traces show the signal intensity at the central peak (3373 G, see Figure 1) as a function of time for different illumination intensities. The LED was turned ON at 2 min (see upright arrow). The approximate PPFD values associated with each LED brightness are labeled.

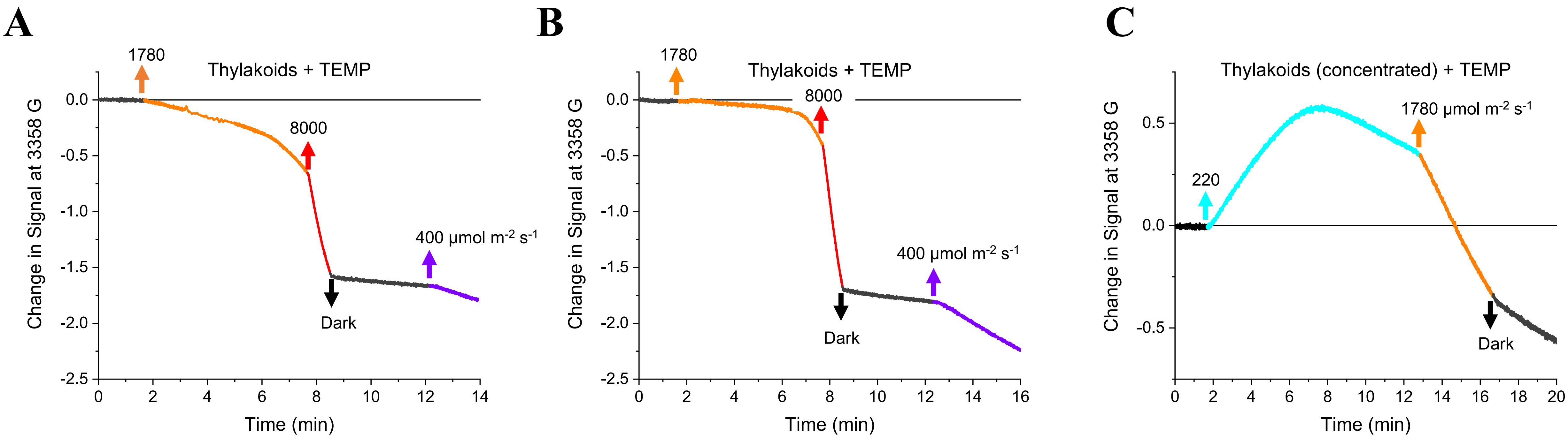


**Figure S6**. Additional replicates for the kinetics of $^1O_2$* in purified thylakoid membranes. Time traces show the change in EPR signal at 3358 G for representative aliquots of thylakoid membrane exposed to changes in illumination intensity inside the EPR cavity. Upward- and downward-facing arrows indicate the timing for light intensity increases and decreases, respectively. Labels specify the approximate light intensity (µmol photons $m^{-2}$ $s^{-1}$). Thylakoid concentration was approximately 50 µg Chl/mL in panels **A,B** and 500 µg Chl/mL in panel **C**. Light-induced increases in EPR signal were only observed for the concentrated thylakoid aliquots (also see **Fig. 3b** in main text). TEMP (2,2,6,6-tetramethypiperidine) were used at a concentration of 50 mM in glycerol resuspension buffer (pH 6) with 0.5 mM ATP.

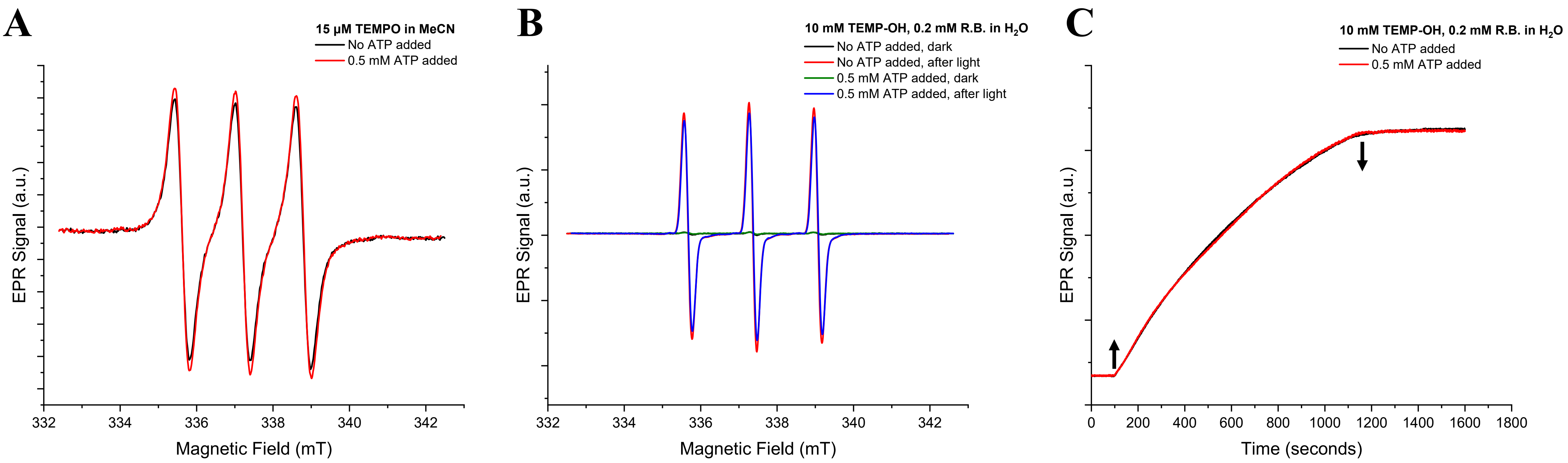


**Figure S7**. EPR control measurements indicate there is no effect of ATP on the EPR nitroxide signal. (**A**) Effect of 0.5 mM ATP on the X-band EPR spectrum of TEMPO (15 μM) in acetonitrile. (**B,C**) Effect of 0.5 mM ATP on the the photoinduced nitroxide radical product from illuminating rose bengal (0.2 mM) in the presence of TEMP-OH (10 mM) in water. Panel B shows the X-band EPR spectra in the presence and absence of ATP. Panel C shows the corresponding kinetic time traces for the same samples shown in Panel B. For panels B and C, illumination of the sample was initiated in the EPR cavity at 100 sec (see upright arrow) using PPFD ~2000 μmol photons $m^{-2}$ $s^{-1}$.

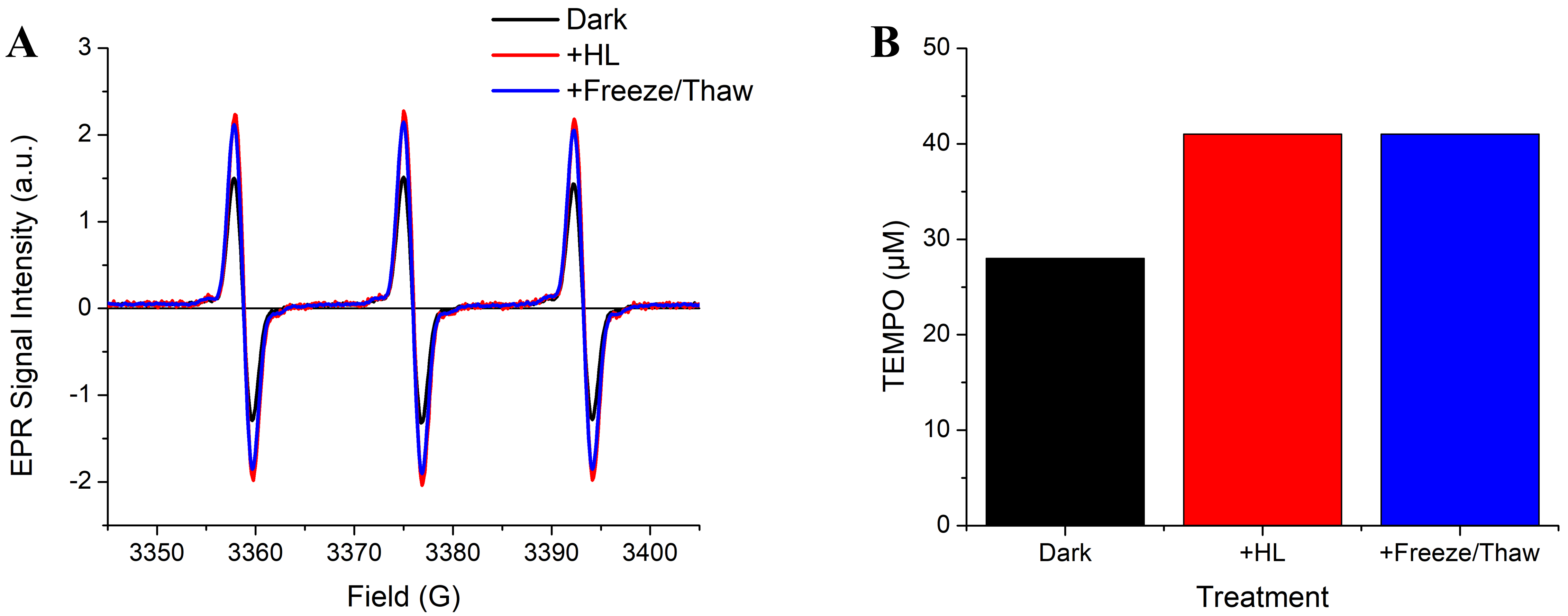


**Figure S8**. Freeze-thaw effect on TEMPO EPR signal. (**A**) EPR spectra for a capillary with thylakoid membranes and TEMP in the dark (*black*), following 100 sec of HL illumination in the EPR cavity (*red*), and after freezing and thawing (*blue*). Note that illumination conditions for this test experiment are slightly different from those described in Figure 5. (**B**) Concentration of TEMPO calculated from each first-derivative EPR spectrum in panel A.

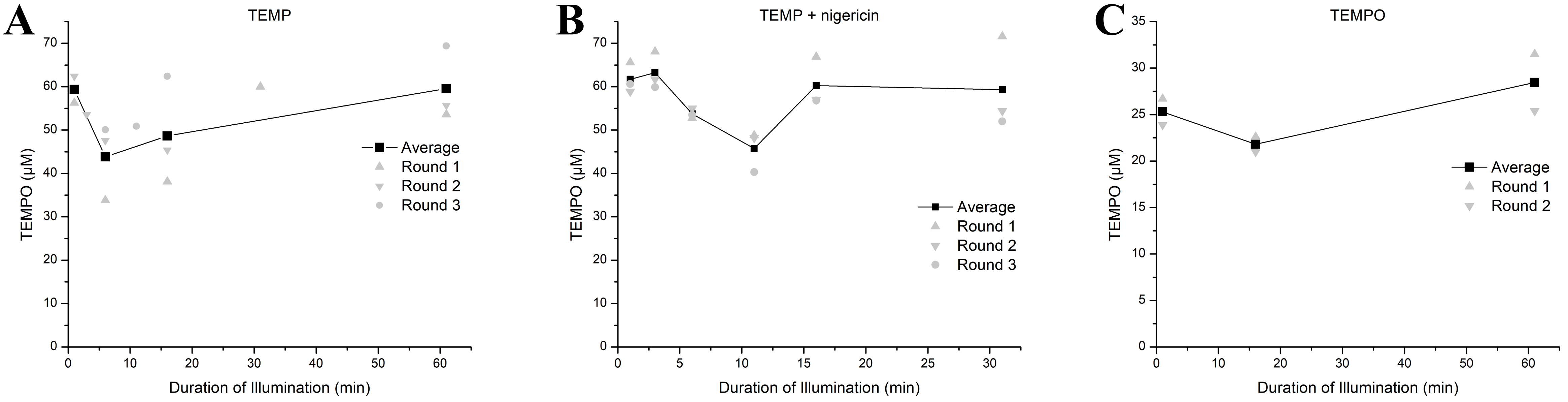


**Figure S9**. (**A**, **B**) Raw data used to generate Fig. 5B, C showing measured concentrations of TEMPO in μM. (**C**) Effect of incubating TEMPO nitroxide radical with a thylakoid membrane. As a control experiment to assess the effect of pre-illumination on the apparent spin concentration, thylakoids were incubated with TEMPO for exactly 1 minute in the light following durations of 0, 15, or 60 minutes of pre-illumination. The concentration of TEMPO measured for all durations of illumination was within ~10% of each other. While it is possible that this small change reflects differences in the concentration or composition of reductants during illumination of membranes, it is more likely representative of the uncertainty (~10%) associated with the EPR measurements especially given that each thylakoid sample was measured using a different capillary. Therefore, the concentration of thylakoid reducing equivalents produced during a short 1-minute incubation is mostly independent of the duration of prior HL illumination of the membranes.

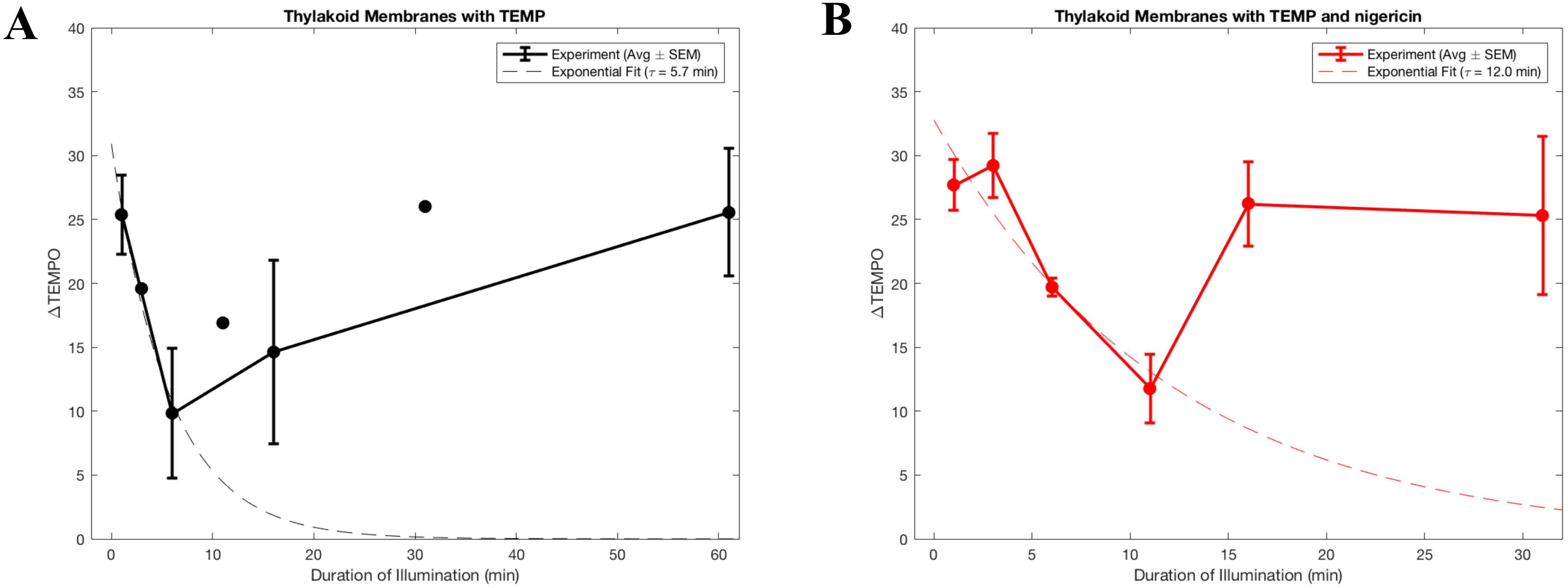


**Figure S10.** Kinetic analysis of changes in TEMPO concentration during illumination of (**A**) untreated and (**B**) nigericin-treated thylakoid membranes. The initial time points were fitted with an exponential to extract the approximate timescale of the decrease in singlet oxygen production, suggesting faster photoprotection in the absence of nigericin (5.7 min vs. 12.0 min). Error bars designate standard error of the mean (n=3).
